# Evolutionary ancestry and novel functions of the mammalian glucose transporter (GLUT) family

**DOI:** 10.1186/1471-2148-10-152

**Published:** 2010-05-21

**Authors:** Amy L Wilson-O'Brien, Nicola Patron, Suzanne Rogers

**Affiliations:** 1 Department of Medicine-St. Vincent's, The University of Melbourne, Fitzroy, Victoria 3065, Australia; 2 Department of Genetics, The University of Melbourne, Parkville, Victoria 3052, Australia; 3 Department of Botany, The University of Melbourne, Parkville, Victoria 3052, Australia; 4 Department of Primary Industries, Victorian Agribiosciences Centre, Bundoora, Victoria 3983, Australia; 5 Protein Chemistry and Metabolism Unit, St. Vincent's Institute of Medical Research, Fitzroy, Victoria 3065, Australia

## Abstract

**Background:**

In general, sugar porters function by proton-coupled symport or facilitative transport modes. Symporters, coupled to electrochemical energy, transport nutrients against a substrate gradient. Facilitative carriers transport sugars along a concentration gradient, thus transport is dependent upon extracellular nutrient levels. Across bacteria, fungi, unicellular non-vertebrates and plants, proton-coupled hexose symport is a crucial process supplying energy under conditions of nutrient flux. In mammals it has been assumed that evolution of whole body regulatory mechanisms would eliminate this need. To determine whether any isoforms bearing this function might be conserved in mammals, we investigated the relationship between the transporters of animals and the proton-coupled hexose symporters found in other species.

**Results:**

We took a comparative genomic approach and have performed the first comprehensive and statistically supported phylogenetic analysis of all mammalian glucose transporter (GLUT) isoforms. Our data reveals the mammalian GLUT proteins segregate into five distinct classes. This evolutionary ancestry gives insight to structure, function and transport mechanisms within the groups. Combined with biological assays, we present novel evidence that, in response to changing nutrient availability and environmental pH, proton-coupled, active glucose symport function is maintained in mammalian cells.

**Conclusions:**

The analyses show the ancestry, evolutionary conservation and biological importance of the GLUT classes. These findings significantly extend our understanding of the evolution of mammalian glucose transport systems. They also reveal that mammals may have conserved an adaptive response to nutrient demand that would have important physiological implications to cell survival and growth.

## Background

Sugars provide energy and structural components to all living cells. Due to their hydrophilic nature, sugars/polyols must first traverse biological lipid bi-layer membranes via carrier-mediated transport mechanisms. Transporter proteins are classified based on phylogenetic and functional data. Within the major facilitator (MF) superfamily the largest group is the sugar porter family [1]. Sugar porters are found in bacteria, archaea and eukarya and function by uniport or proton-coupled symport modes of transport [2]. Uniport, or facilitative sugar carriers, transport a substrate along its concentration gradient, thus transport is dependent upon extracellular nutrient levels. Symporters, or secondary-active transporters, can move a specific nutrient substrate against its concentration gradient in an energy requiring transport process. Energy is sourced from an electrochemical gradient coupling movement of nutrient substrate to translocation of ions.

Bacteria often grow in conditions of extreme environmental flux and therefore secondary-active, energy- dependent symport mechanisms are critical to nutrient supply [3]. The mechanism of sugar accumulation in bacteria is exemplified by the action of the sugar porters of *Escherichia coli *that symport xylose, glucose/galactose or arabinose coupled to proton transfer [4]. For similar reasons proton-coupled sugar symport is also an important process in algae, plants and unicellular eukaryotes [3]. A notable exception to proton-coupled sugar symport in bacteria is the glucose transporter of *Zymonas mobilis *[5]. *Z. mobilis *possesses a facilitative diffusion transport system. This species exists within a plant sap microenvironment associated with high sugar levels, therefore it is likely that the selective pressure to maintain active sugar transport is reduced [6].

Proton-coupled symport of glucose is not known to occur in mammalian tissues. Following food ingestion, endocrine controlled homeostatic processes regulate clearance of blood glucose along its concentration gradient via the facilitative glucose transporter (GLUT) proteins to supply all tissues with fuel. Secondary-active glucose transport does occur in mammals but is mediated by the sodium-coupled glucose co-transporter (SGLT) family members that are distinct at the primary and secondary structural level from the GLUT proteins [7] and are not members of the MF superfamily [1]. Expression of SGLT proteins is primarily restricted to the gut and kidney, where their role is energy-dependent reabsorption of glucose from the lumen [8].

The GLUT transporter family comprises 13 members. Based on sequence, functional and predicted structural similarities the family has been divided into three classes [9]. GLUT transporters exhibit tissue distribution, substrate specificity and transport kinetics that reflects their physiological roles [10]. These however have not been fully defined for all isoforms. The class 1 transporters (GLUT1, 2, 3, and 4) were the first to be identified and have been functionally well described as high affinity facilitative glucose transporters (Additional file 1: Table S1). Class II includes the GLUT5 transporter that has a low affinity for glucose but facilitates the transport of fructose [11]. Recently a novel process whereby another class II transporter GLUT9 exchanges extracellular glucose for intracellular urate has been described [12]. In addition GLUT class III includes by sequence similarity the H^+^-*myo*-inositol transporter, HMIT [9]. Functionally HMIT couples proton translocation to *myo*-inositol symport [13].

**Figure 1:**
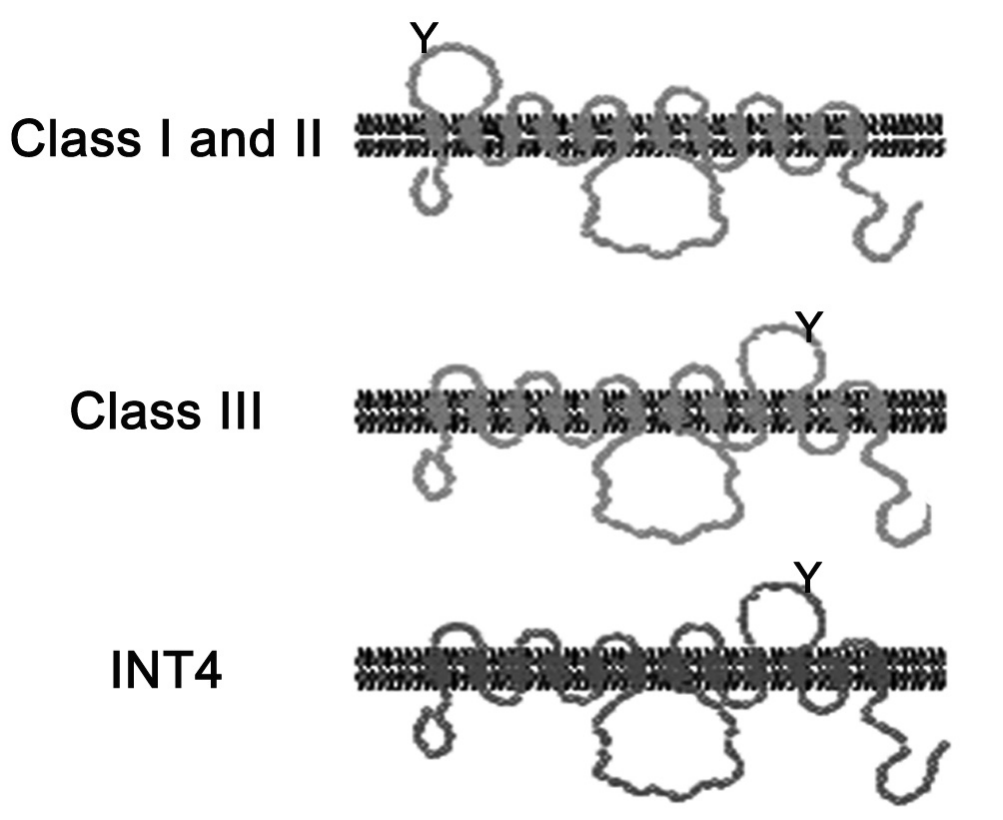
**Predicted sugar transporter membrane topologies**. Shown are the predicted topologies of the class I, II and III GLUT proteins [15], as well as the *A. thaliana *H^+^-*myo*-inositol transporter, AtINT4 [16]. Sites of N-linked glycosylation are indicated (Y).

Significant structural features are conserved between the mammalian glucose facilitators and proton-coupled hexose symporters. GLUT proteins, like all the sugar porter family members, exhibit a predicted twelve transmembrane domain topology [2]. The so-called 'sugar transport signatures' that signify substrate binding and catalytic activity are also conserved across the sugar porter family [2,14]. The recently identified class III group of GLUT proteins exhibit further features highlighting this evolutionary relationship. The membrane topology of the class I and class II GLUT proteins predicts an exofacial loop between the first two helices. This domain contains sites of *N*-linked glycosylation that are associated with functional glucose transport [15]. The domain is not predicted in the class III GLUT structure. Rather, an exofacial loop with sites of *N*-linked glycosylation is predicted between helices 9 and 10 (Figure 1). A similar membrane topology is predicted for proton-coupled sugar symporters in plants [16].

Thus, across bacteria, fungi, unicellular non-vertebrates and plants the mechanism of proton-coupled hexose symport is a crucial process supplying energy under conditions of nutrient flux. By comparison, it has been assumed that the development of whole body regulatory mechanisms in mammals would largely eliminate this need. The precise physiological roles of a number of the more recently identified GLUT molecules are still to be defined [9]. Given the previous studies of sequence similarities and comparable structural characteristics, we questioned whether proton-coupled hexose symport was the ancestral mechanism of sugar transport and whether isoforms with this function have been conserved in mammals.

To address this question we took a comparative genomic approach and have performed the first comprehensive and statistically supported phylogenetic analysis of all the GLUT isoforms and their relatives in non-mammalian species. Our data reveals that the mammalian GLUT proteins segregate into five distinct classes. This evolutionary ancestry gives a clearer insight to the structure and functional relationships of the groups. Combined with biological assays we present evidence that in response to changes in nutrient availability and environmental pH, proton-coupled, active glucose symport has been maintained in mammalian cells. These findings significantly extend our understanding of the evolution of mammalian glucose transport systems. They also reveal that mammals have conserved an adaptive response to nutrient demand that may have important physiological implications to cell survival and growth.

## Results and Discussion

The mammalian GLUT proteins are sugar porter members of the MF superfamily that spans all life forms. We questioned whether proton-coupled glucose symport, common to bacteria, unicellular eukaryotes and plants, could have been maintained as multicellular animals evolved and if not when and why this function was lost. To do this a comparative phylogenomic approach was employed. This strategy is particularly useful to identify new members and novel functions in complex large families [17,18].

Previous evolutionary comparisons of the GLUT proteins have been performed prior to the identification of the complete family and comprised limited sequence similarity alignments. Maiden *et al*. [14] first determined the sequence of the *E. coli *arabinose-H^+ ^and xylose-H^+ ^symporters and reported the seminal finding that both were homologous to the mammalian GLUT1 facilitative transporter. Later, Doege *et al*. [19], suggested that as only one closely related *Drosophila melanogaster *GLUT1 homologue could be identified, the class I transporters may be a specific development in mammals. Based on the sequence similarities of GLUT6 and GLUT8 to bacterial xylose and arabinose-proton symporters, Joost and Thorens [15], originally suggested these transporters represented the oldest GLUT isoforms. Our comprehensive and statistically supported analyses now significantly extend and clarify these comparisons. Sequences of all known mammalian GLUT family members were subject to systematic BLAST searches against the completed genomes of bacteria, fungi, protists, plants, invertebrates and vertebrates (Table 1). Quantitative protein Maximum Likelihood (ML) determinations [20] were used to identify evolutionary relationships. Combined with functional, experimental data five key findings arise.

**Table 1:** Genomes and databases used for molecular phylogenetic analysis


**Metazoans:**	

*Homo sapiens* *Mus musculus* *Gallus gallus* *Drosophila melanogaster* *Caenorhabditis elegans*	http://www.ncbi.nlm.nih.gov/^1^ http://www.ncbi.nlm.nih.gov/^1^ http://www.ncbi.nlm.nih.gov/^1^ http://www.ncbi.nlm.nih.gov/^1^ http://www.ncbi.nlm.nih.gov/^1^

**Fungi:**	

*Saccharomyces cerevisiae* *Schizosaccharomyces pombe* *Aspergillus fumigatus *Af293	http://www.ncbi.nlm.nih.gov/^1^ http://www.ncbi.nlm.nih.gov/^1^ http://www.ncbi.nlm.nih.gov/^1^

**Mycetazoa:**	

*Dictyostelium discoidum*	http://www.ncbi.nlm.nih.gov/^1^

**Archeaplastida:**	

*Arabidopsis thaliana* *Oryza sativa japonica cultivar-group* *Cyanidischiyzon merolae* *Ostreococcus lucimarinus CCE9901*	http://www.arabidopsis.org^2^ http://www.ncbi.nlm.nih.gov/^1^ http://merolae.biol.s.u-tokyo.ac.jp/^3^ http://genome.jgi-psf.org/^4^

**Stramenopiles:**	

*Thalassiosira pseudonana* *Phytophthora sojae*	http://genome.jgi-psf.org/^4^ http://genome.jgi-psf.org/^4^

**Alveolate:**	

*Plasmodium falciparum*	http://www.plasmodb.org/plasmo/home.jsp^5^

**Bacteria:**	

*Escherichia coli *K12 *Streptomyces coelicolor *A3(2) *Mesorhizobium loti* *Bacillus subtilis *str. 168 *Synechocystis sp. *PCC 6803	http://www.ncbi.nlm.nih.gov/^1^ http://www.ncbi.nlm.nih.gov/^1^ http://www.ncbi.nlm.nih.gov/^1^ http://www.ncbi.nlm.nih.gov/^1^ http://http://www.ncbi.nlm.nih.gov/^1^

^1 ^National Centre for Biotechnology Information

^2 ^The Arabidopsis Information Resource

^3 ^*Cyanidioschyzon merolae *Genome Project

^4 ^The Plasmodium Genome Resource

### Comparative genomic analyses reveal five distinct mammalian GLUT classes

**Figure 2:**
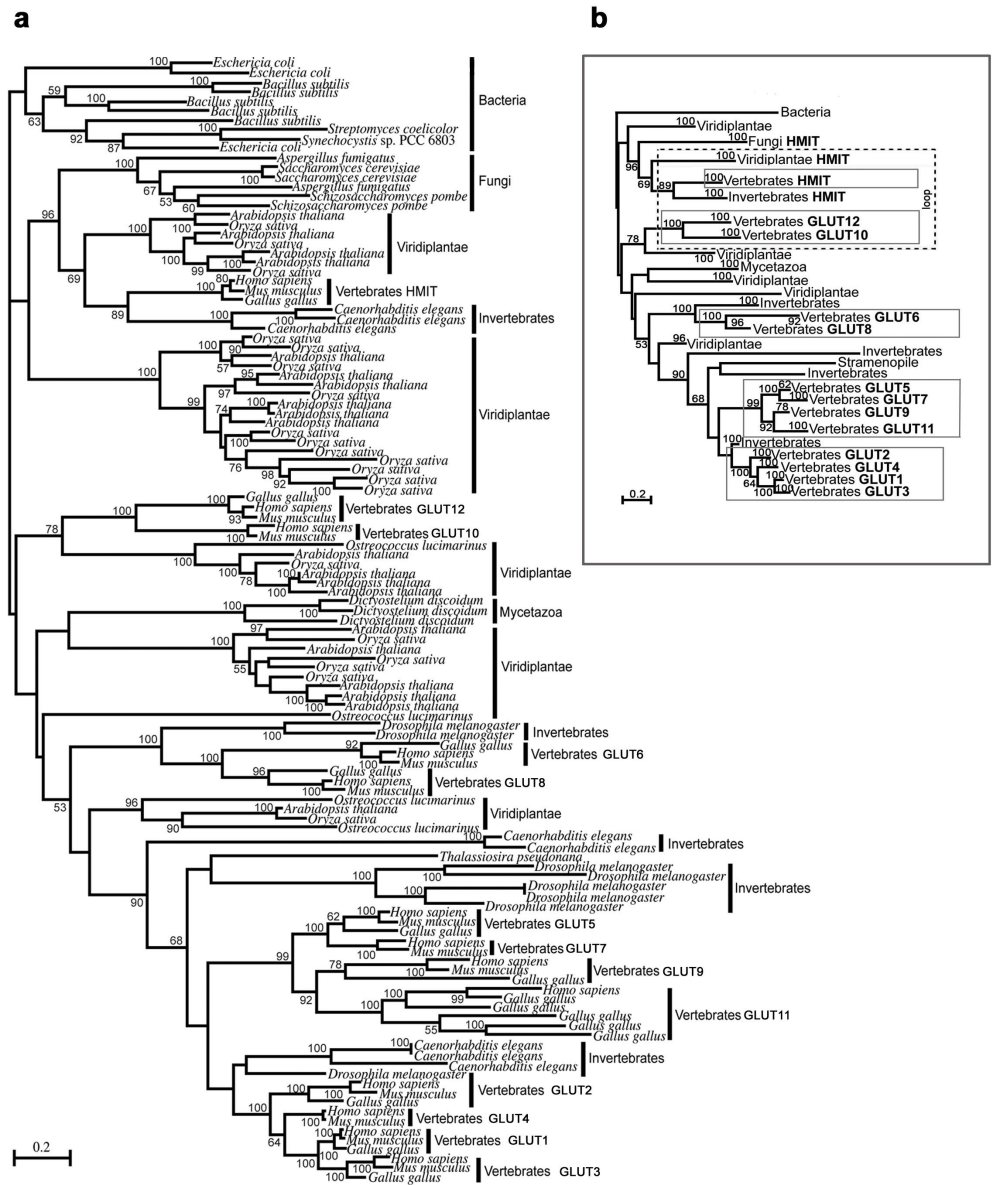
**Phylogenetic analyses of the mammalian GLUT proteins**. **(a) **Human GLUTs 1-12 and HMIT protein sequences were subject to BLAST searches against the complete genomes of select eukaryotes and prokaryotes. Maximum likelihood phylogeny was inferred from the amino acid sequences of transporter proteins following BLAST searches and ClustalX alignment. Bootstrap supports for groups are given at nodes and tips. The complete phylogenetic tree, with expanded tips showing each individual sequence is shown. **(b) **Phylogenetic tree illustrating the evolutionary ancestry of five mammalian GLUT classes. Isoforms exhibiting an extended insertion corresponding to the predicted exofacial loop between helices 9 and 10 are indicated.

Phylogenetic analyses revealed that the class I and class II mammalian GLUT proteins form distinct, well-supported clades. The class I (GLUT1, 2, 3, 4) and class II (GLUT5, 7, 9, 11) proteins resolved as distinct, well-supported (respectively 100% and 99% bootstrap) clades (Figure 2a). Additionally, although support is not strong, class I and class II resolve as sisters. In contrast, we show that the mammalian proteins designated as class III GLUT members (GLUT6, 8, 10, 12 and HMIT) do not form a distinct clade but that the members are dispersed in the evolutionary tree amongst non-mammalian sequences (Figure 2a). GLUT6 and GLUT8 are sisters with strong support (100% bootstrap). GLUT10 and GLUT12 form a distinct clade with strong support (100% bootstrap). HMIT sequences fall with good support (96% bootstrap) within a larger but separate clade of sequences. Thus we find the GLUT proteins designated as class III fall into three separate groups and that there may be five functionally distinct mammalian GLUT classes (Figure 2b).

### Comparative genomic analyses reveal archetypal mammalian GLUT isoforms

Class I and class II GLUT proteins have no non-metazoan relatives, suggesting these orthologues arose relatively recently, after the divergence of multi-cellular animals and are specific to that lineage (Figure 2a). It is noted however, that without sequence information from the unicellular ancestors of animals, this can only be supported by the lack of sampled Opisthokonts (Mycetazoa and Fungi) within these clades. The class I clade contains invertebrate (*D. melanogaster*) sequences branching at the base with strong support (100% bootstrap) indicating the existence of this class in invertebrates. The class II clade contains only vertebrate sequences and may have arisen after the divergence of this phylum. The clade containing GLUT6 and GLUT8 also shows invertebrate (*D. melanogaster*) sequences branching at the base with 100% bootstrap support, suggesting they may have arisen after the divergence of the metazoans, although again this conclusion is limited by available sequence data of earlier-branching species.

In contrast, clades containing GLUT10, GLUT12 and HMIT have sister groups that contain more distantly related taxa (Figure 2a). The mammalian HMIT sequences fall within a large but distinct clade comprised of vertebrate, invertebrate, fungal and plant sequences with good support (96% bootstrap). Many of the members of this clade have been functionally tested (Additional file 1: Figure S1 and Table S1) and show conserved function across all species as H^+^-*myo*-inositol symporters [16,21]. Significantly, though support is only fair (78% bootstrap), the closest relatives of mammalian GLUT10 and GLUT12 are functionally tested or predicted H^+^-glucose symporters of Viridiplantae species (Additional file 1: Table S1 and Figure S1). These include a functionally tested active sugar transporter of *Arabidopsis thaliana *that is thought to couple proton translocation to accumulation of glucose in the plant vacuole [22]. Sequences from fungi and invertebrates did not resolve in this clade, however the presence of sequences from mammals, which diverged later, suggests gene loss in these lineages.

The tree suggests that more than one GLUT isoform existed in ancestral eukaryotes before the divergence of the three hypothetical supergroups (Opsthokonta, Chromalveolata and Archeaplastida) for which genome data is available. As the backbone of the tree is not well supported it cannot be said which of GLUT10, GLUT12 or HMIT was the most ancestral, or which might have been duplicated to give rise to the class I and class II GLUT isoforms in metazoans. Overall, the tree did not reject the theory that proton-coupled glucose symport could be a function of mammalian GLUT genes.

Therefore, to further pursue our hypothesis that proton-coupled glucose symport could be conserved in mammals, we then conducted a series of experiments using the mammalian GLUT12 facilitative glucose transporter, of which the closest non-animal relatives were shown to be proton-coupled hexose symporters of Viridiplantae (Additional file 1: Table S1 and Figure S1). We now demonstrate that in response to changes in nutrient availability and environmental pH, proton-coupled, active glucose symport function is maintained in mammalian cells.

### Up-hill, pH dependent glucose transport is maintained in mammalian cells

We have previously demonstrated GLUT12-mediated, facilitative glucose transport by exogenous expression in *Xenopus laevis *oocytes, a system that is useful to define transporter function due to low endogenous glucose transport levels [23]. We have also shown in a novel assay using polarized epithelial cells and neutralizing antibodies, that in high glucose conditions GLUT12 is targeted to the apical membrane of Madin Darby Canine Kidney (MDCK) epithelial cells, where it mediates facilitative glucose transport [24]. In this assay, cells are retrovirally transfected with a GLUT12 cDNA containing a c-Myc epitope tag in the fifth exofacial loop (GLUT12myc) and glucose transport (as a function of glucose analogue uptake) assayed at the apical surface. By comparing transport in the presence of either neutralizing α-c-Myc or unrelated control α-hemagglutinin (HA) antibodies, we can specifically determine GLUT12-mediated glucose transport [24]. To test whether under conditions where intracellular glucose levels exceed those in the external environment GLUT12 can also mediate up-hill glucose transport, we have now employed this model for vectorial analysis of glucose transport activity.

**Figure 3:**
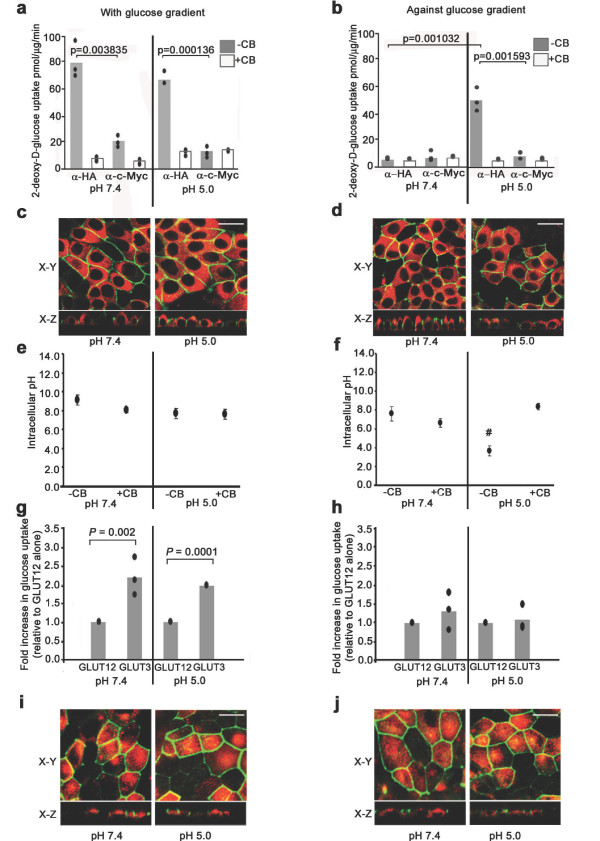
**Effects of pH and glucose gradients on GLUT12-mediated glucose transport**. **(a) **Facilitative transport occurs down a glucose gradient at pH 7.4 and pH 5.0. **(b) **Active glucose transport occurs against a glucose gradient at pH 5.0 only. Representative experiments are shown. Bars represent mean 2-deoxy-D-glucose uptake with data point range plotted (n = 3). *P *values < 0.05 were considered significant and are shown on the graphs. **(c, d) **Immunofluoresence detection demonstrates apical localization of endogenous GLUT12 under all conditions used for glucose uptake assays (X-Y and X-Z planes shown). GLUT12, red; polarization of MDCK cells was determined by immunofluorescent labelling of the tight junction marker ZO-1, green. Bar, 20 μm. **(e, f) **Flow cytometry recorded intracellular pH of MDCK cells under conditions used in glucose uptake assays. Mean and SD were calculated (n = 6). A representative experiment is shown. # Significant decrease in pH compared to all other treatment groups. *P *= 0.0001. **(g) **Glucose uptake assays were performed in MDCK cells expressing endogenous GLUT12 and retrovirally transfected GLUT3. 2-deoxy-D-glucose transport with a glucose gradient at both pH 7.4 and pH 5.0 was significantly increased in MDCK cells retrovirally expressing GLUT3, in comparison to MDCK cells expressing endogenous GLUT12 only. **(h) **In cells retrovirally expressing GLUT3 no significant increase in 2-deoxy-D-glucose transport against a glucose gradient at pH 7.4 or pH 5.0 was recorded. Representative experiments are shown. Bars represent fold increase in 2-deoxy-D-glucose uptake with data point range plotted (n = 3). *P *values of < 0.05 were considered significant and are shown on the graphs. **(i, j) **Immunofluorescence detection of retrovirally transfected GLUT3 at the apical membrane of MDCK cells under conditions used for glucose uptake assays. GLUT3, red; tight junctional marker ZO-1, green. Bar, 20 μm.

Transport of labelled glucose analogue was assayed at the apical membrane of polarized MDCK cells retrovirally transfected with GLUT12myc. Transport was assayed with or against glucose concentration gradients at pH 7.4 or 5.0, in the presence of either neutralizing α-c-Myc or control α-HA antibodies (Figure 3). At both neutral pH and in acidic conditions, GLUT12 facilitated glucose transport with a glucose gradient into MDCK cells (Figure 3a). Facilitative transport was inhibited by cytochalasin B, a specific inhibitor of glucose transport proteins. Glucose gradients were then established by pre-incubation in high (25 mM) glucose and immediately replacing the incubation media with low (5 mM) glucose, so that the extracellular glucose concentration would be lower than the intracellular concentration. At pH 7.4, GLUT12 did not mediate transport against a glucose gradient (Figure 3b). At pH 5.0, GLUT12 mediated significant transport against a glucose gradient (Figure 3b). GLUT12-mediated transport against a glucose gradient into MDCK cells was blocked by cytochalasin B. We have previously demonstrated that the subcellular distribution of both endogenously expressed GLUT12 and GLUT12myc are regulated in MDCK cells by chronic (greater than 16 hours) exposure to changes in extracellular glucose levels [24]. We therefore confirmed that the differences in glucose transport observed were not due to altered GLUT12 localization in response to changes in pH or acute glucose flux (Figure 3c, d).

We have also tested the ability of the class I facilitator GLUT3 to mediate uphill glucose transport. We, and others have previously shown that when expressed in MDCK cells GLUT3, like GLUT12, is targeted to the apical membrane [24,25]. Therefore, glucose transport at the apical membrane of MDCK cells stably expressing GLUT3 was compared to that of non-transfected cells. In GLUT3 transfected cells, a significant increase in glucose transport rate (above that attributable to transport via endogenously expressed GLUT12) was measured under conditions designed to create an inward but not outward glucose gradient (Figure 3g, h). Under all experimental conditions confocal immunofluorescence confirmed GLUT3 localization to the apical cell surface (Figure 3i, j). In addition, we have performed glucose transport assays in 3T3-L1 fibroblasts where we demonstrate glucose transport with, but not against, a glucose gradient (Additional file 1: Figure S2). 3T3-L1 fibroblasts express the class I glucose transporter GLUT1 but do not express GLUT12 [26]. The results suggest that GLUT12 but not the class I facilitators GLUT1 or GLUT3 is able to transport glucose uphill, against its concentration gradient.

There are limitations to the experimental design used. We cannot exclude that glucose loading to create an outward facing gradient could induce a *trans*-stimulation effect. *Trans*-stimulation has been described as the effect of intracellular glucose binding to the facilitative transporter molecule in the inward facing conformation, stimulating re-orientation to an outward facing conformer, and increasing apparent transport. The phenomenon is particularly evident in isolated membrane vesicle preparations [27,28] and has also been described in analysis of an active transport mechanism in plants [29]. Additionally, the glucose phosphorylation rate in MDCK cells is not known. MDCK cells are derived from the epithelium of renal distal tubules [30] and high expression levels of hexokinase have been reported in distal portions of the nephron [31]. Thus, we could not exclude the possibility that a reverse hexose gradient may not have been maintained under the experimental conditions used.

### GLUT12 mediates proton-coupled, glucose symport

Therefore to further investigate whether the pH dependence of GLUT12-mediated, up-hill glucose transport represents proton-coupled glucose symport, we used flow cytometry to correlate H^+^-driven change in intracellular pH with transport against a glucose concentration gradient. Cells loaded with the pH sensitive dye BCECF/AM (the acetoxymethylester form of 2',7'-bis(carboxyethyl)-5(6)-carboxyfluorescein) were incubated under conditions used for glucose uptake assays. Intracellular pH was calibrated (Additional file 1: Figure S3). No change in intracellular pH was recorded in conditions of facilitative glucose transport (Figure 3e). In contrast, we find a significant decrease in intracellular pH correlates to conditions of up-hill glucose transport, suggesting GLUT12-mediated active glucose transport occurs via a mechanism of H^+^-coupled glucose symport. Consistent with this finding, intracellular acidification was inhibited when cytochalasin B was added to block glucose transport against a glucose gradient, thus linking glucose and proton transport (Figure 3f).

**Figure 4:**
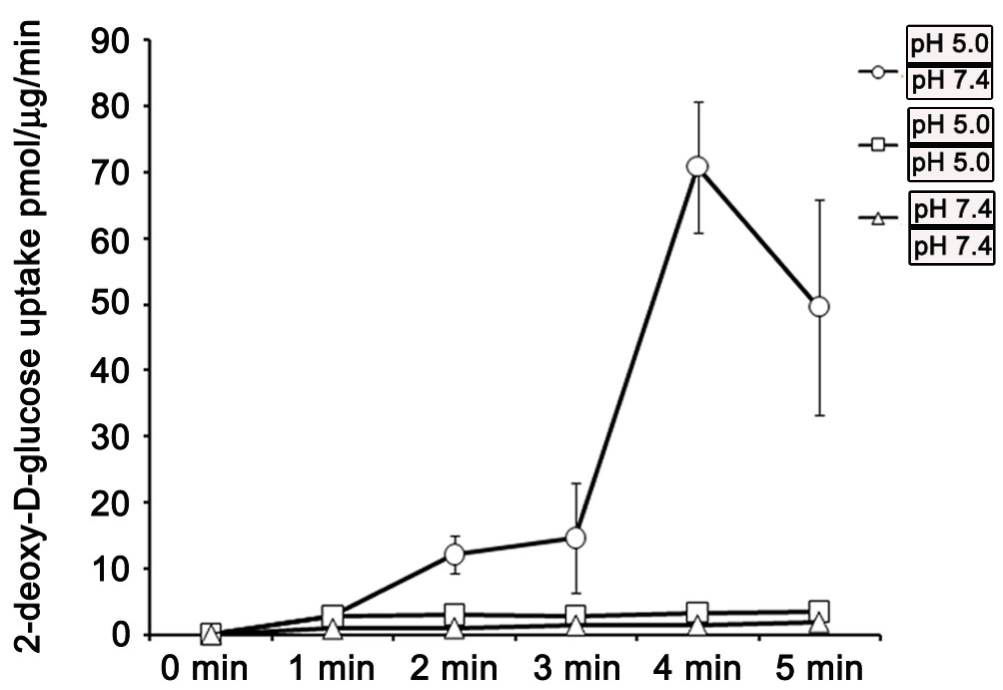
**Effects of membrane H^+ ^gradients on glucose analogue accumulation**. MDCK cells were preloaded with glucose to establish outward facing glucose gradients. Accumulation of glucose tracer was then measured in the presence or absence of an inward directed H^+ ^gradient as indicated (pH extracellular = 5.0; pH intracellular = 7.4), (pH extracellular = 5.0; pH intracellular = 5.0) (pH extracellular = 7.4; pH intracellular = 7.4). Triplicate accumulation time-points samples were measured. Mean and SD were calculated. A representative experiment is shown.

We next performed experiments to determine whether low extracellular pH energized uphill, GLUT12-mediated glucose transport by means of an inward directed proton gradient across the apical membrane. To do this polarized MDCK cells were again preloaded with glucose. However in these experiments cells were preloaded with glucose at either neutral or acid pH and accumulation of glucose (as a function of glucose analogue) measured. Intracellular accumulation of glucose analogue against a glucose gradient only occurred when an inward directed H^+ ^gradient (pH extracellular = 5.0; pH intracellular = 7.4) was present (Figure 4). In contrast when the electrochemical gradient was disrupted (pH intracellular = pH extracellular), no accumulation of glucose analogue was recorded at either pH 5.0 or pH 7.4. The experimental design differentiates between the direct effects of external pH itself upon glucose transport and the requirement for an electrochemical gradient [32]. In combination our experiments show that under conditions designed to create an outward facing glucose gradient, glucose transport is pH dependent, is inhibited by cytochalasin B, requires the presence of an inward facing electrochemical gradient and results in intra-cellular acidification. The results support our interpretation that GLUT12 can mediate proton-coupled, glucose symport.

### GLUT12 is not a myo-inositol transporter

**Figure 5:**
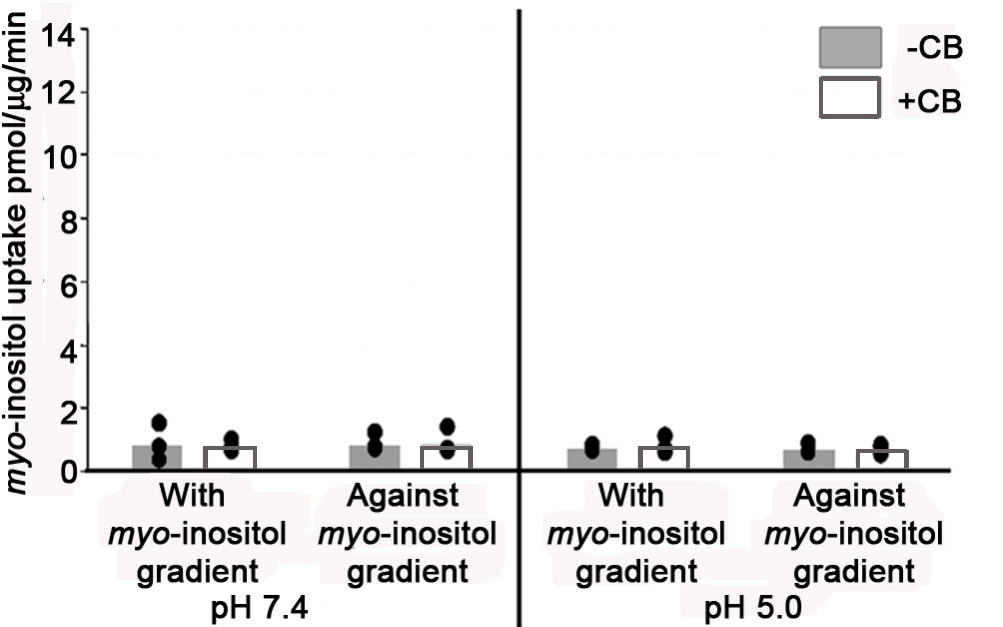
**GLUT12 does not mediate *myo*-inositol transport**. *Myo*-inositol transport assays were performed as described in materials and methods. No significant *myo*-inositol uptake was observed in MDCK cells at either pH 7.4 or pH 5.0. Representative experiments are shown. Bars represent mean *myo*-inositol uptake with data point range plotted (n = 3).

GLUT10, GLUT12 and HMIT of vertebrates and HMIT of invertebrates and plants contain an extended insertion at position 371-466 (relative to the human GLUT12 protein sequence) corresponding to the predicted exofacial loop between helices 9 and 10 (Figure 1 and Figure 2b). Although potential sites of N-linked glycosylation are present, this insertion is truncated in GLUT6 and GLUT8, further supporting their classification as a separate class (Figure 2b). Uldrey et al. [13] noted conserved and potential functionally related motifs within the exofacial loop of HMIT. We find the sequence identity of the insertion is conserved within HMIT of fungi, plants and animals; notably several cysteine residues are conserved. However, the identity of the insertion sequence is not conserved between HMIT and vertebrate GLUT10 or GLUT12. Lack of sequence conservation between the predicted exofacial loops of GLUT12 and HMIT therefore suggests that *myo*-inositol is not a GLUT12 substrate and their distinct positions in separate well-supported clades supports the interpretation. To functionally test this, we assayed for *myo*-inositol transport at the apical surface of polarized MDCK cells. No significant *myo*-inositol transport was detected. Assays were performed both with or against a *myo*-inositol substrate gradient, and at pH 5.0 and 7.4 (Figure 5). Control *myo*-inositol transport assays were also performed (Additional file 1: Figure S4) in the neuroblastoma cell line SH-SY5Y that express the Na^+^-*myo*-inositol transporter [33].

## Conclusions

Our analyses support the hypothesis that proton-coupled symport is the ancestral mechanism of sugar transport. Close relatives of HMIT, GLUT10 and GLUT12 are present in extant eukaryotic lineages. GLUT classes I and II as well as GLUT6 and GLUT8 appear to be specific to multicellular animals. HMIT, GLUT10 and GLUT12 retain similarities to transporters of single-celled organisms and plants that actively transport hexoses and may have retained this function when hexoses are limiting or at equilibrium. Our experiments suggest the class I isoforms GLUT3 and GLUT1 may not exhibit this activity. However it is not known whether all isoforms outside of HMIT, GLUT12 and GLUT10 would have lost the ability to undergo proton-coupled hexose symport. As shown in Additional file 1: Figure S1 and Table S1, symport activity has been inferred for a number invertebrate transporters closely related to the mammalian class II and GLUT6 and GLUT8 transporters.

That mammals may have conserved an adaptive response to nutrient demand could have important physiological implications to cell survival and growth. GLUT12 facilitates glucose transport in nutrient excess; in nutrient demand, GLUT12 could allow intracellular accumulation of glucose above extracellular levels. GLUT12 is expressed in epithelial breast and prostate tumors that exhibit elevated glycolytic metabolism and lactate production, resulting in an acidic environment [34,35]. Glycolytic metabolism of glucose is thought to initiate in response to hypoxic conditions, early in tumor formation before a tumor vascular network has been laid down and continues throughout tumor development. To produce sufficient ATP via glycolysis rather than aerobic metabolism, cancer cells require more glucose and over-expression of glucose transporter proteins is also frequently found [34,35]. GLUT12-mediated glucose symport could therefore be significant to both the initiation and progression of tumorigenesis by providing energy for cancerous growth, particularly where blood vessel networks and thus glucose supply are poor.

## Methods

### Evolutionary analyses

Genomes (Table 1) were subject to BLAST analysis with human GLUTs 1-12 and HMIT sequences. Sequences with an e-value of less than 10^5 ^were used in reciprocal BLAST searches and did not identify any sequences other than GLUTs 1-12 and HMIT. The sequences below the cut-off were aligned by ClustalX [36]. Sequences that compromised the inclusion set due to either large deletions or unalignable sections were removed due to the limitations of phylogenetic analysis. The resulting matrix contained 191 taxa, all having a BLAST scores over 0.0001 and 333 unambiguously aligned characters. This matrix was subjected to neighbor-joining analysis by WEIGHBOR with 4 rate categories [37]. Rates and invariable sites were calculated by PUZZLE [38]. Alignments are available on request. Alignments were subject to maximum likelihood (ML) analysis by PhyML [20] with a WAG substitution matrix and 9 rate categories. The α value was 1.909 and number of invariable sites 0.011. Bootstrap resampling was performed with PUZZLEBOOT [38]. 100 bootstrap trees were calculated by PhyML. Parameters were as for ML analysis but with 4 gamma correction categories.

### Materials and cell culture

Media was obtained from Invitrogen (Carlsbad, CA, USA) and fetal bovine serum (FBS) from JRH Bioscience (Lenexa, KS, USA). Where used, media was acidified with HCl on the day of use. Rabbit polyclonal α-GLUT12 antibody has been described [39]. Other antibodies used were obtained as follows: α-c-Myc (clone 9E10) and α-hemagglutinin (HA) (Santa Cruz Biotechnology, Santa Cruz, CA, USA); α-ZO-1 (Zonula Occludens - 1) (Zymed, San Francisco, CA, USA); α-GLUT3 (Abcam, Cambridge, MA, USA); Fluorescein isothiocyanate (FITC)-conjugated (Dako, Carpinteria, CA, USA); Alexa-568-conjugated (Molecular Probes, Carlsbad, CA, USA). All other reagents were obtained from Sigma (St. Louis, MO, USA) unless otherwise stated. MDCK and 3T3-L1 cell lines were maintained in 25 mM glucose Dulbecco's Modified Eagle Medium (DMEM)/5% FBS. MDCK cells were grown for 3-4 days in DMEM/10% FBS to a polarized monolayer. SH-SY5Y cells were maintained in Roswell Park Memorial Institute Medium (RPMI)/15% FBS. The GLUT12myc and GLUT3 retroviral constructs and MDCK retroviral transfection procedures have been previously described [24].

### Glucose transport assays

Transport assays and the use of either neutralizing α-c-Myc or control α-HA antibodies were performed as previously described [24]. Briefly, as GLUT12 is targeted to the apical plasma membrane in high glucose conditions, in assays with a glucose gradient, cells were equilibrated for 30 minutes with equal volumes of 25 mM glucose DMEM and 2× (glucose free) uptake solution (final extracellular concentration 12.5 mM glucose). Glucose transport assays were initiated by the addition of 2× uptake solution (either pH 7.4 or 5.0) containing 25 mM cold 2-D-deoxyglucose (to create an inward facing gradient with a final total extracellular hexose concentration of 18.75 mM) and 1 μCi/ml 2-[1,2-3H(N)]deoxy-d-glucose (PerkinElmer, Boston, MA, USA). In assays against a glucose gradient, cells were equilibrated in 25 mM glucose DMEM for 30 minutes; immediately prior to glucose transport assay extracellular equilibration media was quickly replaced with 5 mM glucose DMEM. Glucose transport assays were then initiated by the addition of 2× uptake solution (either pH 7.4 or 5.0) containing 5 mM cold 2-D-deoxyglucose (to create an outward facing hexose gradient with a final total extracellular hexose concentration of 5 mM) and 1 μCi/ml 2-[1,2-3H(N)]deoxy-d-glucose. To measure intracellular accumulation of glucose against a gradient, transport assays were performed against a glucose gradient at specified pH, with assays being terminated at one-minute intervals, up to five minutes. SH-5YSY cells were grown to 90% confluence in 6 well dishes. Assays of *myo*-inositol transport were performed as described [13,33]. For *myo*-inositol transport assays in MDCK monolayers, cells were polarized in 12 well dishes and assayed as described above with modifications: 200 μM *myo*-inositol and 1 μCi/ml inositol, *myo*-[2-3H(N)] (Perkin Elmer) was used in the 2× uptake solution.

### Flow cytometry

MDCK cells were polarized in 12 well dishes and equilibrated as for transport assays. When required, acidified media was added immediately prior to flow cytometry. Uptake buffer containing 1.5 μM BCECF/AM was added for 5 min. BCECF loaded cells were trypsinized, centrifuged and resuspended in PBS. Samples were analyzed at 488 and 568 nm with a FACSCalibur flow cytometer (BD Biosciences, San Jose, CA, USA) counting 10,000 events. The signal was calibrated by incubating BCECF loaded cells for 5 min with 10 μM nigericin (Merck, Darmstadt, Germany) in high K^+ ^buffer (pH 6.0 - 8.0 in 0.25 increments) [40]. Data was analyzed with BD CellQuest Pro software (BD Biosciences).

### Immunofluorescence

Confocal, immunofluorescence detection and protein localization were performed as described in Wilson-O'Brien et al., 2008 [24]. A Bio-Rad MCR1024 confocal microscope system (Bio-Rad, Hercules, CA, USA), mounted to a Leica DM112B inverted microscope (Leica, Germany) was used to determine GLUT12 and GLUT3 protein localization in MDCK cells. A 60X APO/1.32 oil immersion lens (Leica) was used. Excitation of FITC was provided by the 488 nm of a Krypton/Argon laser with the laser power set at 10% of maximum. Excitation of AlexaFluor568 was provided by 568 nm of a Krypton/Argon laser with the laser power set at 10% of maximum. Fluorescence was collected using a 585-680 nm emission filter. Images were collected using Bio-Rad Laser-Sharp software (Bio-Rad). The resulting images were analysed using Confocal Assistant ver. 4.02, and Adobe Photoshop was used to generate all figures.

### Statistical analyses

Experiments were performed at least 3 times (glucose uptakes, n = 3; flow cytometry, n = 6, for each treatment). Data was analyzed by one-way ANOVA and unpaired t-tests.

## Abbreviations

BCECF/AM: the acetoxymethylester form of 2',7'-bis(carboxyethyl)-5(6)-carboxyfluorescein; DMEM: Dulbecco's modified eagle medium; FBS: fetal bovine serum; FITC: fluorescein isothiocyanate; GLUT: glucose transporter; GLUT12myc: GLUT12 cDNA containing a c-Myc epitope tag in the fifth exofacial loop; HMIT: H^+^-*myo*-inositol transporter; HA: α-hemagglutinin; MDCK: Madin Darby canine kidney; MF: major facilitator, ML: Maximum Likelihood; RPMI: Roswell Park Memorial Institute medium; SGLT: Na^+^-coupled glucose transporter; ZO-1: Zonula occludens - 1.

## Authors' contributions

AWOB was responsible for experimental work and data analysis. NP performed the molecular evolutionary analysis and interpretation. SR, with the help of the other authors, was responsible for project concept, design, data analysis and writing. All authors read and approved the final manuscript.

## Supplementary Material

Additional file 1 **Supplementary Table 1 - Accession numbers and functional annotations of sequences used in this study**. Numbers refer to annotated tree in Supplementary Figure 1. **Supplementary Figure 1 - Phylogenetic analyses of the mammalian GLUT proteins**. Numbers refer to the annotations in Supplementary Table 1. **Supplementary Figure 2 - Effects of pH and glucose gradients on glucose transport in 3T3-L1 fibroblasts**. Glucose transport occurs with, but not against, a glucose gradient. **Supplementary Figure 3 - Representative flow cytometry calibration curve**. Calibration of the BCECF/AM signal was performed by incubation of BCECF loaded MDCK cells in high potassium buffers of known pH. **Supplementary Figure 4 - *Myo*-inositol uptake by the neuroblastoma cell line SH-SY5Y**. Transport of *myo*-inositol in SY5Y cells was used as a positive control for the MDCK *myo*-inositol uptake assays. Click here for file
