# Supplementary material for: Evolutionary ancestry and novel functions of the mammalian glucose transporter (GLUT) family
Source: BMC Evol Biol. 2010 May 21;10:152. doi: 10.1186/1471-2148-10-152 (PMC2890515; doi:10.1186/1471-2148-10-152)
Supplement: Additional file 1 — Supplementary Table 1 - Accession numbers and functional annotations of sequences used in this study. Numbers refer to annotated tree in Supplementary Figure 1. Supplementary Figure 1 - Phylogenetic analyses of the mammalian GLUT proteins. Numbers refer to the annotations in Supplementary Table 1. Supplementary Figure 2 - Effects of pH and glucose gradients on glucose transport in 3T3-L1 fibroblasts. Glucose transport occurs with, but not against, a glucose gradient. Supplementary Figure 3 - Representative flow cytometry calibration curve. Calibration of the BCECF/AM signal was performed by incubation of BCECF loaded MDCK cells in high potassium buffers of known pH. Supplementary Figure 4 - Myo-inositol uptake by the neuroblastoma cell line SH-SY5Y. Transport of myo-inositol in SY5Y cells was used as a positive control for the MDCK myo-inositol uptake assays. [file 1471-2148-10-152-S1.PDF]

**Supplementary Table 1 - Accession numbers (NCBI: National Centre for Biotechnology Information) and functional annotations of sequences used in this study. Numbers refer to annotated tree in Supplementary figure 1.**

| Number | Species | Acc# | Annotation | Functional Study |
|---|---|---|---|---|
| 1 | *Escherichia coli* | NP_417418 | GalP; D-galactose transporter; symporter activity | Riordan and Kornberg, 1977 |
| 2 | *Escherichia coli* | NP_289394 | AraE; Arabinose transporter; symporter activity | Maiden et al, 1988 |
| 3 | *Bacillus subtilis* | NP_391276 | AraE; Arabinose transporter ; symporter activity | Krispin O, Allmansberger R., 1998 |
| 4 | *Bacillus subtilis* | NP_388707 | YfiG; Putative sugar transporter | |
| 5 | *Bacilus subtilis* | CAB07473 | YwtG; Putative metabolite transport protein | |
| 6 | *Bacilus subtilis* | NP_391860 | CsbC; Putative sugar transporter | |
| 7 | *Bacilus subtilis* | NP_388504 | IolT; Myo-inositol transporter | Yoshida et al, 2002 |
| 8 | *Streptomyces coelicolor* | NP_628802 | Transmembrane efflux protein | |
| 9 | *Synechocystis sp. PCC 6803* | NP_442047 | Glucose transport protein | |
| 10 | *Escherichia coli* | NP_290665 | XylE; D-xylose transporter; symporter activity | Lam et al, 1980 Sumiya et al, 1995 |
| 11 | *Aspergillus fumigatus* | XP_755126 | Putative myo-inositol transporter | |
| 12 | *Saccharomyces cerevisiae* | NP_010785 | Itr1p; Myo-inositol transporter | Nikawa et al., 1991 |
| 13 | *Saccharomyces cerevisiae* | NP_014538 | Itr2p; Myo-inositol transporter | Nikawa et al., 1991 |
| 14 | *Aspergillus fumigatus* | XP_752216 | Myo-inositol transporter | |
| 15 | *Schizosaccharomyces pombe* | NP_593320 | Myo-inositol transporter | Niederberger et al, 1998 |
| 16 | *Schizosaccharomyces pombe* | NP_593858 | Itr1; Myo-inositol transporter | Niederberger et al, 1998 |
| 17 | *Arabidopsis thaliana* | NP_850393 | INT1; Inositol transporter 1; symporter activity | Schneider et al, 2008 |
| 18 | *Oryza sativa* | NP_001053172 | Hypothetical protein | |
| 19 | *Arabidopsis thaliana* | NP_174313 | INT2; Inositol transporter 2; symporter | Schneider et al, 2007 |

| | | | | |
|---|---|---|---|---|
| | | | activity | |
| 20 | *Oryza sativa* | NP_001058902 | Putative myo-inositol transporter | |
| 21 | *Arabidopsis thaliana* | NP_181117 | INT3; Inositol transporter 3; inferred symporter activity | |
| 22 | *Arabidopsis thaliana* | NP_193381 | INT4; Inositol transporter 4; symporter activity | Schneider et al, 2006 |
| 23 | *Oryza sativa* | NP_001053292 | Hypothetical protein | |
| 24 | *Homo sapiens* | NP_443117 | HMIT; H(+)-myo-inositol cotransporter; symporter activity | Uldry et al, 2001 |
| 25 | *Mus musculus* | NP_001028805 | HMIT; H(+)-myo-inositol cotransporter; symporter activity | |
| 26 | *Gallus gallus* | XP_001232940 | HMIT; predicted H(+)-myo-inositol cotransporter; inferred symporter activity | |
| 27 | *Caenorhabditis elegans* | NP_507623 | HMIT1.1; H(+)MyoInositol coTransporter family member ; inferred symporter activity | |
| 28 | *Caenorhabditis elegans* | NP_507624 | HMIT1.2; H(+)MyoInositol coTransporter family member; inferred symporter activity | |
| 29 | *Caenorhabditis elegans* | NP_497725 | HMIT1.3; H(+)MyoInositol coTransporter family member; inferred symporter activity | |
| 30 | *Oryza sativa* | NP_001054276 | Hypothetical protein | |
| 31 | *Oryza sativa* | NP_001054277 | Hypothetical protein | |
| 32 | *Arabidopsis thaliana* | NP_179671 | Putative mannitol transporter; inferred symporter activity | |
| 33 | *Oryza sativa* | NP_001053383 | Hypothetical protein | |
| 34 | *Arabidopsis thaliana* | NP_179438 | Putative mannitol transporter; inferred symporter activity | |
| 35 | *Arabidopsis thaliana* | NP_195385 | Putative mannitol transporter; inferred symporter activity | |
| 36 | *Oryza sativa* | AAK13147 | Putative sugar transporter | |
| 37 | *Arabidopsis thaliana* | NP_179210 | Mannitol transporter, putative; inferred symporter activity | |
| 38 | *Arabidopsis thaliana* | NP_179209 | Putative mannitol transporter; inferred | |

| | | | | |
|---|---|---|---|---|
| | | | symporter activity | |
| 39 | *Arabidopsis thaliana* | NP_188513 | ATPLT5; polyol transporter 5; symporter activity | Klepek et al, 2005 |
| 40 | *Oryza sativa* | NP_001060108 | Putative sugar transporter | |
| 41 | *Oryza sativa* | NP_001041938 | Putative sugar transporter | |
| 42 | *Oryza sativa* | NP_001049269 | Putative mannitol transporter | |
| 43 | *Oryza sativa* | NP_001066872 | Sugar transporter family protein | |
| 44 | *Oryza sativa* | NP_001066878 | Putative sugar transporter | |
| 45 | *Oryza sativa* | NP_001068334 | Putative sugar transporter | |
| 46 | *Oryza sativa* | NP_001068332 | Putative sugar transporter | |
| 47 | *Gallus gallus* | XP_419733 | Similar to facilitative glucose transporter | |
| 48 | *Homo sapiens* | NP_660159 | GLUT12; solute carrier family 2 member 12 | Rogers et al, 2003 |
| 49 | *Mus musculus* | BAC29262 | GLUT12; solute carrier family 2 member 12 | |
| 50 | *Homo sapiens* | NP_110404 | GLUT10; solute carrier family 2 member 10 | Dawson et al, 2001 |
| 51 | *Mus musculus* | NP_569718 | GLUT10; solute carrier family 2 member 10 | |
| 52 | *Ostreococcus lucimarinus* | XP_001416926 | MFS family transporter | |
| 53 | *Arabidopsis thaliana* | NP_200733 | Sugar transporter family protein; inferred symporter activity | |
| 54 | *Oryza sativa* | NP_001065505 | Putative sugar transporter | |
| 55 | *Arabidopsis thaliana* | NP_172214 | ATSTP2; Sugar transporter 2; symporter activity | Truernit et al, 1999 |
| 56 | *Arabidopsis thaliana* | NP_197203 | Sugar transporter family protein; inferred symporter activity | |
| 57 | *Arabidopsis thaliana* | NP_186959 | AtVGT1; vacuolar glucose transporter; symporter activity | Aluri and Buttner, 2007 |
| 58 | *Dictyostelium discoideum* | XP_629391 | Calmodulin binding protein | Catalano and O’Day, 2007 |
| 59 | *Dictyostelium discoideum* | XP_642498 | Sugar transporter family protein | |
| 60 | *Dictyostelium discoideum* | XP_640848 | Sugar transporter family protein | |
| 61 | *Arabidopsis thaliana* | NP_200960 | Putative hexose transporter; inferred symporter activity | |
| 62 | *Oryza sativa* | NP_001065385 | Putative hexose transporter; inferred symporter activity | |
| 63 | *Arabidopsis thaliana* | NP_172592 | STP1; sugar transporter 1; symporter activity | Sauer et al, 1990 |

| | | | | |
|---|---|---|---|---|
| 64 | *Oryza sativa* | NP_001045970 | Putative monosaccharide transporter; inferred symporter activity | |
| 65 | *Oryza sativa* | NP_001043369 | Putative monosaccharide transporter | |
| 66 | *Oryza sativa* | NP_001058704 | MST1; Putative monosaccharide transport protein | |
| 67 | *Arabidopsis thaliana* | NP_197718.1 | STP11; sugar transporter 11**;** symporter activity | Schneidereit et al, 2005 |
| 68 | *Arabidopsis thaliana* | NP_188628 | Putative monosaccharide transporter; inferred symporter activity | |
| 69 | *Arabidopsis thaliana* | NP_175449 | STP9; Sugar transporter 9; symporter activity | Schneidereit et al, 2003 |
| 70 | *Ostreococcus lucimarinus* | XP_001418575 | MFS family transporter | |
| 71 | *Drosophila melanogaster* | NP_611234 | CG6484; Sugar transporter | |
| 72 | *Drosophila melanogaster* | NP_523675 | Sut4; Sugar transporter 4 , isoform A; inferred symporter activity | |
| 73 | *Gallus gallus* | XP_423637 | GLUT6; Predicted solute carrier family 2, member 6 | |
| 74 | *Homo sapiens* | NP_060055 | GLUT6; Solute carrier family 2, member 6 | Kayano et al, 1990 |
| 75 | *Mus musculus* | NP_766247 | GLUT6; solute carrier family 2, member 6 | |
| 76 | *Gallus gallus* | NP_989706 | GLUT8; Predicted solute carrier family 2, member 8 | |
| 77 | *Homo sapiens* | NP_055395 | GLUT8; solute carrier family 2, member 8 | Doege et al, 2000 |
| 78 | *Mus musculus* | NP_062361 | GLUT8; solute carrier family 2, member 8 | Carayannopoulos et al ,2000 |
| 79 | *Ostreococcus lucimarinus* | XP_001422705 | MFS family hexose transporter | |
| 80 | *Arabidopsis thaliana* | NP_568328 | PGLCT; Plastidic glucose translocator; symporter activity | Weber at al, 2000 |
| 81 | *Oryza sativa* | NP_001041938 | Sugar transporter | |
| 82 | *Ostreococcus lucimarinus* | XP_001417096 | MFS family transporter | |
| 83 | *Caenorhabditis elegans* | NP_504430 | Hypothetical protein | |
| 84 | *Caenorhabditis elegans* | NP_506734 | Hypothetical protein | |
| 85 | *Thalassiosira pseudonana* | XP_002291581 | Sugar transporter | |
| 86 | *Drosophila melanogaster* | NP_524732 | Sut2; Sugar transporter 2; inferred symporter activity | |
| 87 | *Drosophila* | NP_524731 | Sut3; Sugar transporter | |

| | | | | |
|---|---|---|---|---|
| | *melanogaster* | | 3; inferred symporter activity | |
| 88 | *Drosophila melanogaster* | NP_724438 | CG7882, isoform A; MFS family transporter | |
| 89 | *Drosophila melanogaster* | NP_610189 | CG7882, isoform B; MFS family transporter | |
| 90 | *Drosophila melanogaster* | NP_523658 | Sut1; Sugar transporter 1; inferred symporter activity | |
| 91 | *Homo sapiens* | NP_003030 | GLUT5; solute carrier family 2, member 5 | Kayano at al, 1990 |
| 92 | *Mus musculus* | NP_062715 | GLUT5; Solute carrier family 2, member 5 | Corpe et al, 2002 |
| 93 | *Gallus gallus* | XP_417596.2 | GLUT5; Predicted solute carrier family 2, member 5 | |
| 94 | *Homo sapiens* | NP_997303 | GLUT7; Solute carrier family 2, member 7 | Li et al, 2004 |
| 95 | *Mus musculus* | NP_001078998 | GLUT7; Solute carrier family 2, member 7 | |
| 96 | *Homo sapiens* | NP_001001290 | GLUT9; Solute carrier family 2 member 9 | Doege et al, 2000 |
| 97 | *Gallus gallus* | XP_420789 | GLUT9; Predicted solute carrier family 2, member 9 | |
| 98 | *Gallus gallus* | XP_426528 | Predicted sugar transporter | |
| 99 | *Homo sapiens* | NP_110434 | GLUT11; Solute carrier family 2, member 11 | Doege et al, 2001 |
| 100 | *Gallus gallus* | XP_425279 | GLUT11; Predicted solute carrier family 2, member 11 | |
| 101 | *Gallus gallus* | XP_415207 | Hypothetical protein | |
| 102 | *Gallus gallus* | XP_415227 | Hypothetical protein | |
| 103 | *Gallus gallus* | XP_001232864 | Hypothetical protein | |
| 104 | *Gallus gallus* | XP_001232846 | Hypothetical protein | |
| 105 | *Caenorhabditis elegans* | NP_493982 | Hypothetical protein | |
| 106 | *Caenorhabditis elegans* | NP_493981 | Hypothetical protein | |
| 107 | *Caenorhabditis elegans* | NP_503413 | Hypothetical protein | |
| 108 | *Drosophila melanogaster* | NP_523878 | Dmel/GLUT1; Glucose transporter 1, isoform B | |
| 109 | *Homo sapiens* | NP_000331 | GLUT2; solute carrier family 2 member 2 | Gould et al 1991 |
| 110 | *Mus musculus* | NP_112474 | GLUT2; Solute carrier family 2, member 2 | |
| 111 | *Gallus gallus* | NP_997061 | GLUT2; Predicted solute carrier family 2, member 2 | |
| 112 | *Homo sapiens* | NP_001033 | GLUT4; Solute carrier family 2, member 4 | Keller and Mueckler, 1990 |
| 113 | *Mus musculus* | NP_033230 | GLUT4; Solute carrier family 2, member 4 | |

| | | | | |
|---|---|---|---|---|
| 114 | *Homo sapiens* | AAH94735 | GLUT1; Solute carrier family 2, member 1 | Keller and Mueckler, 1990 |
| 115 | *Mus musculus* | NP_001090573 | GLUT1; Solute carrier family 2 member 1 | |
| 116 | *Gallus gallus* | NP_990540 | GLUT1; Predicted solute carrier family 2, member 1 | |
| 117 | *Homo sapiens* | NP_008862 | GLUT3; Solute carrier family 2, member 3 | Collville et al, 1993 |
| 118 | *Mus musculus* | NP_035531 | GLUT3; Solute carrier family 2, member 3 | |
| 119 | *Gallus gallus* | NP_990842 | GLUT3; Predicted solute carrier family 2 member 3 | |


Aluri S, Buttner M (2007) Identification and functional expression of the Arabidopsis thaliana vacuolar glucose transporter 1 and its role in seed germination and flowering. *Proc Natl Acad Sci U S A* **104:** 2537-2542

Carayannopoulos MO, Chi MM, Cui Y, Pingsterhaus JM, McKnight RA, Mueckler M, Devaskar SU, Moley KH (2000) GLUT8 is a glucose transporter responsible for insulin-stimulated glucose uptake in the blastocyst. *Proc Natl Acad Sci U S A* **97:** 7313-7318

Catalano A, O'Day DH (2008) Calmodulin-binding proteins in the model organism Dictyostelium: a complete & critical review. *Cell Signal* **20:** 277-291

Colville CA, Seatter MJ, Jess TJ, Gould GW, Thomas HM (1993) Kinetic analysis of the liver-type (GLUT2) and brain-type (GLUT3) glucose transporters in Xenopus oocytes: substrate specificities and effects of transport inhibitors. *Biochem J* **290 ( Pt 3):** 701-706

Corpe CP, Bovelander FJ, Munoz CM, Hoekstra JH, Simpson IA, Kwon O, Levine M, Burant CF (2002) Cloning and functional characterization of the mouse fructose transporter, GLUT5. *Biochim Biophys Acta* **1576:** 191-197

Dawson PA, Mychaleckyj JC, Fossey SC, Mihic SJ, Craddock AL, Bowden DW (2001) Sequence and functional analysis of GLUT10: a glucose transporter in the Type 2 diabetes-linked region of chromosome 20q12-13.1. *Molecular genetics and metabolism* **74:** 186-199

Doege H, Bocianski A, Joost HG, Schurmann A (2000a) Activity and genomic organization of human glucose transporter 9 (GLUT9), a novel member of the family of sugar-transport facilitators predominantly expressed in brain and leucocytes. *Biochem J* **350 Pt 3:** 771-776

Doege H, Bocianski A, Scheepers A, Axer H, Eckel J, Joost HG, Schurmann A (2001) Characterization of human glucose transporter (GLUT) 11 (encoded by SLC2A11), a novel sugar-transport facilitator specifically expressed in heart and skeletal muscle. *Biochem J* **359:** 443-449

Doege H, Schurmann A, Bahrenberg G, Brauers A, Joost HG (2000b) GLUT8, a novel member of the sugar transport facilitator family with glucose transport activity. *J Biol Chem* **275:** 16275-16280

Gould GW, Thomas HM, Jess TJ, Bell GI (1991) Expression of human glucose transporters in Xenopus oocytes: kinetic characterization and substrate specificities of the erythrocyte, liver, and brain isoforms. *Biochemistry* **30:** 5139-5145

Kayano T, Burant CF, Fukumoto H, Gould GW, Fan YS, Eddy RL, Byers MG, Shows TB, Seino S, Bell GI (1990) Human facilitative glucose transporters. Isolation, functional characterization, and gene localization of cDNAs encoding an isoform (GLUT5) expressed in small intestine, kidney, muscle, and adipose tissue and an unusual glucose transporter pseudogene-like sequence (GLUT6). *J Biol Chem* **265:** 13276-13282

Keller K, Mueckler M (1990) Different mammalian facilitative glucose transporters expressed in Xenopus oocytes. *Biomedica biochimica acta* **49:** 1201-1203

Klepek YS, Geiger D, Stadler R, Klebl F, Landouar-Arsivaud L, Lemoine R, Hedrich R, Sauer N (2005) Arabidopsis POLYOL TRANSPORTER5, a new member of the monosaccharide transporter-like superfamily, mediates H+-Symport of numerous substrates, including myo-inositol, glycerol, and ribose. *Plant Cell* **17:** 204-218

Krispin O, Allmansberger R (1998) The Bacillus subtilis AraE protein displays a broad substrate specificity for several different sugars. *J Bacteriol* **180:** 3250-3252

Lam VM, Daruwalla KR, Henderson PJ, Jones-Mortimer MC (1980) Proton-linked D-xylose transport in Escherichia coli. *J Bacteriol* **143:** 396-402

Li Q, Manolescu A, Ritzel M, Yao S, Slugoski M, Young JD, Chen XZ, Cheeseman CI (2004) Cloning and Functional Characterization of the Human GLUT7 Isoform (SLC2A7) from the Small Intestine. *Am J Physiol Gastrointest Liver Physiol*

Maiden MC, Jones-Mortimer MC, Henderson PJ (1988) The cloning, DNA sequence, and overexpression of the gene araE coding for arabinose-proton symport in Escherichia coli K12. *J Biol Chem* **263:** 8003-8010

Niederberger C, Graub R, Schweingruber AM, Fankhauser H, Rusu M, Poitelea M, Edenharter L, Schweingruber ME (1998) Exogenous inositol and genes responsible for inositol transport are required for mating and sporulation in Shizosaccharomyces pombe. *Curr Genet* **33:** 255-261

Nikawa J, Tsukagoshi Y, Yamashita S (1991) Isolation and characterization of two distinct myo-inositol transporter genes of Saccharomyces cerevisiae. *J Biol Chem* **266:** 11184-11191

Riordan C, Kornberg HL (1977) Location of galP, a gene which specifies galactose permease activity, on the Escherichia coli linkage map. *Proc R Soc Lond B Biol Sci* **198:** 401-410

Rogers S, Chandler JD, Clarke AL, Petrou S, Best JD (2003) Glucose transporter GLUT12-functional characterisation in *Xenopus laevis* oocytes. *Biochem Biophys Res Commun* **308:** 422-426

Sauer N, Friedlander K, Graml-Wicke U (1990) Primary structure, genomic organization and heterologous expression of a glucose transporter from Arabidopsis thaliana. *EMBO J* **9:** 3045-3050

Schneider S, Beyhl D, Hedrich R, Sauer N (2008) Functional and physiological characterization of Arabidopsis INOSITOL TRANSPORTER1, a novel tonoplast-localized transporter for myo-inositol. *Plant Cell* **20:** 1073-1087

Schneider S, Schneidereit A, Konrad KR, Hajirezaei MR, Gramann M, Hedrich R, Sauer N (2006) Arabidopsis INOSITOL TRANSPORTER4 mediates high-affinity H+ symport of myoinositol across the plasma membrane. *Plant Physiol* **141:** 565-577

Schneider S, Schneidereit A, Udvardi P, Hammes U, Gramann M, Dietrich P, Sauer N (2007) Arabidopsis INOSITOL TRANSPORTER2 mediates H+ symport of different inositol epimers and derivatives across the plasma membrane. *Plant Physiol* **145:** 1395-1407

Schneidereit A, Scholz-Starke J, Buttner M (2003) Functional characterization and expression analyses of the glucose-specific AtSTP9 monosaccharide transporter in pollen of Arabidopsis. *Plant Physiol* **133:** 182-190

Schneidereit A, Scholz-Starke J, Sauer N, Buttner M (2005) AtSTP11, a pollen tube-specific monosaccharide transporter in Arabidopsis. *Planta* **221:** 48-55

Sumiya M, Davis EO, Packman LC, McDonald TP, Henderson PJ (1995) Molecular genetics of a receptor protein for D-xylose, encoded by the gene xylF, in Escherichia coli. *Receptors Channels* **3:** 117-128

Truernit E, Stadler R, Baier K, Sauer N (1999) A male gametophyte-specific monosaccharide transporter in Arabidopsis. *Plant J* **17:** 191-201

Uldry M, Ibberson M, Horisberger JD, Chatton JY, Riederer BM, Thorens B (2001) Identification of a mammalian H(+)-myo-inositol symporter expressed predominantly in the brain. *EMBO J* **20:** 4467-4477

Weber A, Servaites JC, Geiger DR, Kofler H, Hille D, Groner F, Hebbeker U, Flugge UI (2000) Identification, purification, and molecular cloning of a putative plastidic glucose translocator. *Plant Cell* **12:** 787-802

Yoshida K, Yamamoto Y, Omae K, Yamamoto M, Fujita Y (2002) Identification of two myo-inositol transporter genes of Bacillus subtilis. *J Bacteriol* **184:** 983-991

Figure S1

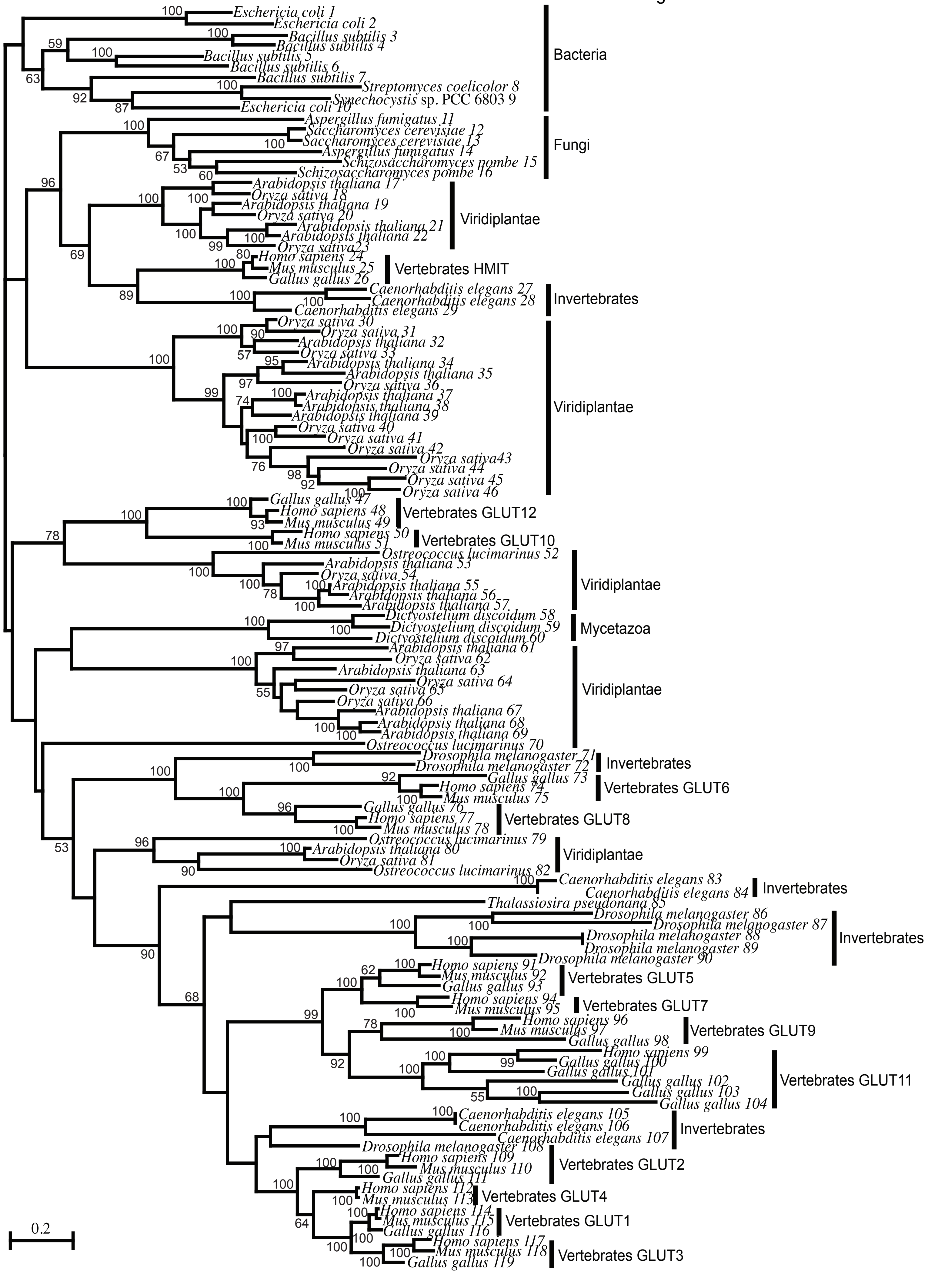
Eschericia coli 1
Eschericia coli 2
Bacillus subtilis 3
Bacillus subtilis 4
Bacillus subtilis 5
Bacillus subtilis 6
Bacillus subtilis 7
Streptomyces coelicolor 8
Synechocystis sp. PCC 6803 9
Eschericia coli 10
Bacteria
Aspergillus fumigatus 11
Saccharomyces cerevisiae 12
Saccharomyces cerevisiae 13
Aspergillus fumigatus 14
Schizosaccharomyces pombe 15
Schizosaccharomyces pombe 16
Fungi
Arabidopsis thaliana 17
Oryza sativa 18
Arabidopsis thaliana 19
Oryza sativa 20
Arabidopsis thaliana 21
Arabidopsis thaliana 22
Oryza sativa23
Viridiplantae
Homo sapiens 24
Mus musculus 25
Gallus gallus 26
Vertebrates HMIT
Caenorhabditis elegans 27
Caenorhabditis elegans 28
Caenorhabditis elegans 29
Invertebrates
Oryza sativa 30
Oryza sativa 31
Arabidopsis thaliana 32
Oryza sativa 33
Arabidopsis thaliana 34
Arabidopsis thaliana 35
Oryza sativa 36
Arabidopsis thaliana 37
Arabidopsis thaliana 38
Arabidopsis thaliana 39
Oryza sativa 40
Oryza sativa 41
Oryza sativa 42
Oryza sativa43
Oryza sativa 44
Oryza sativa 45
Oryza sativa 46
Viridiplantae
Gallus gallus 47
Homo sapiens 48
Mus musculus 49
Vertebrates GLUT12
Homo sapiens 50
Mus musculus 51
Vertebrates GLUT10
Ostreococcus lucimarinus 52
Arabidopsis thaliana 53
Oryza sativa 54
Arabidopsis thaliana 55
Arabidopsis thaliana 56
Arabidopsis thaliana 57
Viridiplantae
Dictyostelium discoidum 58
Dictyostelium discoidum 59
Dictyostelium discoidum 60
Mycetazoa
Arabidopsis thaliana 61
Oryza sativa 62
Arabidopsis thaliana 63
Oryza sativa 64
Oryza sativa 65
Oryza sativa 66
Arabidopsis thaliana 67
Arabidopsis thaliana 68
Arabidopsis thaliana 69
Viridiplantae
Ostreococcus lucimarinus 70
Drosophila melanogaster 71
Drosophila melanogaster 72
Invertebrates
Gallus gallus 73
Homo sapiens 74
Mus musculus 75
Vertebrates GLUT6
Gallus gallus 76
Homo sapiens 77
Mus musculus 78
Vertebrates GLUT8
Ostreococcus lucimarinus 79
Arabidopsis thaliana 80
Oryza sativa 81
Ostreococcus lucimarinus 82
Viridiplantae
Caenorhabditis elegans 83
Caenorhabditis elegans 84
Invertebrates
Thalassiosira pseudonana 85
Drosophila melanogaster 86
Drosophila melanogaster 87
Drosophila melanogaster 88
Drosophila melanogaster 89
Drosophila melanogaster 90
Invertebrates
Homo sapiens 91
Mus musculus 92
Gallus gallus 93
Vertebrates GLUT5
Homo sapiens 94
Mus musculus 95
Vertebrates GLUT7
Homo sapiens 96
Mus musculus 97
Gallus gallus 98
Vertebrates GLUT9
Homo sapiens 99
Gallus gallus 100
Gallus gallus 101
Gallus gallus 102
Gallus gallus 103
Gallus gallus 104
Vertebrates GLUT11
Caenorhabditis elegans 105
Caenorhabditis elegans 106
Caenorhabditis elegans 107
Drosophila melanogaster 108
Invertebrates
Homo sapiens 109
Mus musculus 110
Gallus gallus 111
Vertebrates GLUT2
Homo sapiens 112
Mus musculus 113
Vertebrates GLUT4
Homo sapiens 114
Mus musculus 115
Gallus gallus 116
Vertebrates GLUT1
Homo sapiens 117
Mus musculus 118
Gallus gallus 119
Vertebrates GLUT3
0.2

Figure S2

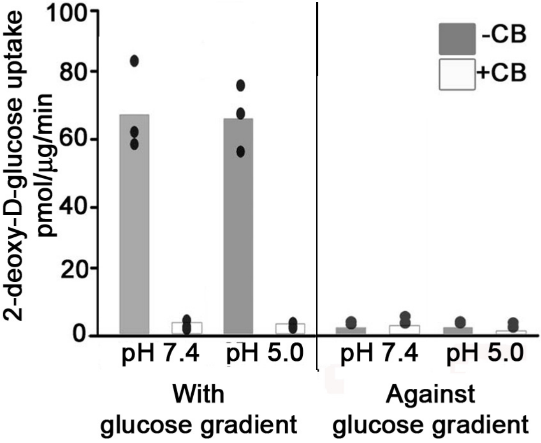

2-deoxy-D-glucose uptake
pmol/µg/min
100
80
60
40
20
0
-CB
+CB
pH 7.4
pH 5.0
pH 7.4
pH 5.0
With
glucose gradient
Against
glucose gradient

Figure S3

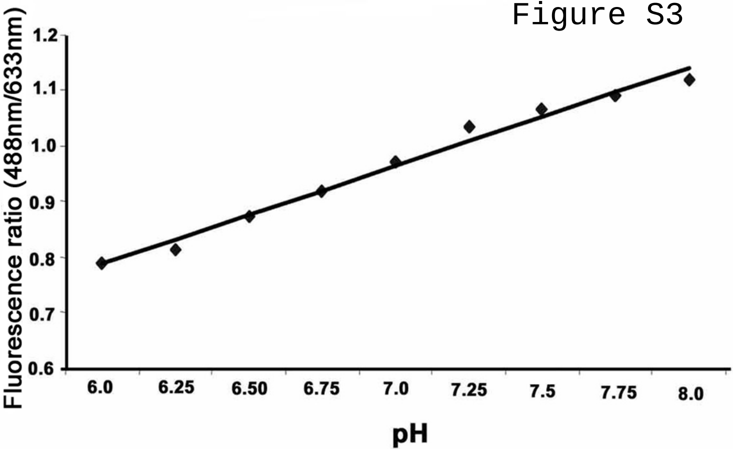

Fluorescence ratio (488nm/633nm)
1.2
1.1
1.0
0.9
0.8
0.7
0.6
6.0
6.25
6.50
6.75
7.0
7.25
7.5
7.75
8.0
pH

# Figure S4

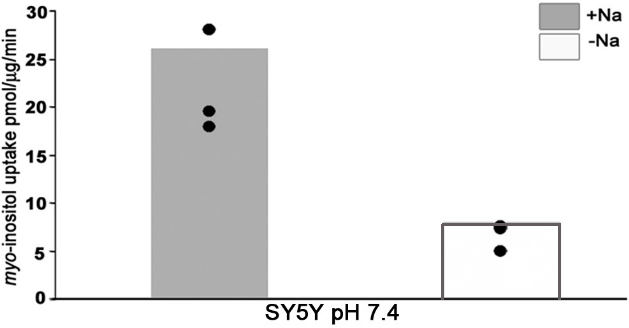

+Na
-Na
*myo*-inositol uptake pmol/μg/min
30
25
20
15
10
5
0
SY5Y pH 7.4
